# Unmixing EEG Inverse Solutions Based on Brain Segmentation

**DOI:** 10.3389/fnins.2018.00325

**Published:** 2018-05-15

**Authors:** Rolando J. Biscay, Jorge F. Bosch-Bayard, Roberto D. Pascual-Marqui

**Affiliations:** ^1^Probabilidad y Estadística, Centro de Investigación en Matemáticas, Guanajuato, Mexico; ^2^Departamento de Neurobiología Conductual y Cognitiva, Instituto de Neurobiología, Universidad Nacional Autónoma de México, Querétaro, Mexico; ^3^The KEY Institute for Brain-Mind Research, University Hospital of Psychiatry, Zurich, Switzerland

**Keywords:** EEG inverse solutions, EEG inverse low resolution, EEG connectivity, unmixing, source connectivity

## Abstract

Due to its low resolution, any EEG inverse solution provides a source estimate at each voxel that is a mixture of the true source values over all the voxels of the brain. This mixing effect usually causes notable distortion in estimates of source connectivity based on inverse solutions. To lessen this shortcoming, an unmixing approach is introduced for EEG inverse solutions based on piecewise approximation of the unknown source by means of a brain segmentation formed by specified Regions of Interests (ROIs). The approach is general and flexible enough to be applied to any inverse solution with any specified family of ROIs, including point, surface and 3D brain regions. Two of its variants are elaborated in detail: arbitrary piecewise constant sources over arbitrary regions and sources with piecewise constant intensity of known direction over cortex surface regions. Numerically, the approach requires just solving a system of linear equations. Bounds for the error of unmixed estimates are also given. Furthermore, insights on the advantages and of variants of this approach for connectivity analysis are discussed through a variety of designed simulated examples.

## Introduction

The physical and mathematical peculiarities of the (ill-posed) EEG inverse problem imposes that any EEG inverse solution unavoidably has low resolution, as have been profusely studied by many authors (see, e.g., Hämäläinen and Ilmoniemi, 1994; Pascual Marqui, 1999; Pascual-Marqui, 2002; Grech et al., 2008; Pascual-Marqui et al., 2011; and references therein). More specifically, the resulting current density (or source) estimate at any given voxel is a mixture of source values over all the voxels of the brain. The coefficients or weights of such mixture are determined by the so-called resolution matrix of the inverse solution.

This “mixing” (or “smoothing”) effect imposes artificial dependences between source estimates at different voxels that are spurious regarding the real dependence structure of the source associated with the underlying neurophysiological activity. Consequently, connectivity estimates based on inverse solutions can severely deviate from the real connectivity patterns.

In general, due to the ill-posed mathematical nature of the EEG inverse problem, without further specific assumptions it is not possible unmixing an EEG inverse solution to recover the true source and its covariance structure for the full set of voxels in the cortex.

On the other hand, for many purposes in functional brain imaging a quite common practice is to approximate the source image by means of a piecewise function regarding several suitably specified regions, usually referred to as Regions of Interest (ROIs) (see, e.g., Daunizeau et al., 2004; Lapalme et al., 2006; Chowdhury et al., 2013), and references therein, for source estimation based on such parcellations of the source space. These approaches incorporate a distributed inverse solution by integrating a parcellation as a form of regularization, but they do not explicitly carry out leakage correction, i.e., unmixing. However, Farahibozorg et al. (2018) introduced adaptive cortical parcellation algorithms for optimizing the number, size and locations of parcels. The resolution matrices of these adaptive parcels showed higher sensitivity to distinguish parcels than standard anatomical parcellations of the brain, allowing the algorithms to minimize the false leakage-induced connections between the regions, produced by the inverse methods, which is also analyzed in that paper.

The central goal of the present paper is to develop a general and flexible approach for unmixing EEG inverse solutions in which such kind of piecewise approximation be applied to the unknown source field. Generality and flexibility is here thought of regarding to allow ROIs of different dimensions and forms, any linear inverse solution, and the feasibility of incorporating additional information about the source when deemed convenient (e.g., a known direction of the source at each voxel of a region). In contrast to other approaches, the unmixing method introduced is a two-step procedure in which a distributed inverse solution step (such as sLORETA) is followed by an unmixing step for leakage correction. Selecting sLoreta for the illustration purpose of this work, does not compromise our algorithm to this method of inverse solution. sLoreta is selected because it is simple, fast to calculate and very popular (at the time we write this work, the Loreta family has more than 5,000 citations in the literature of the inverse solutions methods). Therefore, it will be easier to understand for the many people that uses any method of the Loreta family. It is true that other inverse methods have better resolution than sLoreta and more resistance to noise (like eLoreta, for example), but sLoreta provided the simplicity and performance necessary to illustrate the need and advantages of our proposal.

For this, a natural requirement is that the resulting unmixed source estimate matches the true source when the assumed piecewise approximation is exactly satisfied. On this basis, it is shown that the goal just mentioned can be accomplished by just solving some systems of linear equations.

This approach is general enough to allow for ROIs of any dimension, such as point, surface and 3D brain regions. Two of its variants are elaborated in detail: arbitrary piecewise constant sources over arbitrary regions and sources with piecewise constant intensity of known direction over cortex surface regions. In case in which the adopted piecewise source model holds only as a simplifying approximation, bounds on the error of unmixed source estimates are given. However, due to the nature of the inverse problem, the ROIs cannot be selected in an absolutely arbitrary way if an identifiable model is desired. There are limitations in the number and configuration of the ROIs that will be theoretically studied in the body of this work, being the number of ROIs restricted by the number of electrodes.

Advantages and limitations of the approach are explored and discussed through a number of enlightening simulated examples. In particular, it is shown that the introduced unmixing procedure is more robust to model misspecifications that direct fitting of parceled models.

## Mixing effects in source estimation

Some notations will be introduced to be used throughout this paper. **v** denotes the *d*×*1* vector of scalp electric potentials (multichannel EEG) measured at some instant over a number *d* of electrodes on the scalp. *U* = {*u* : *u* ∈ {1, …, *N*_*vox*_} is a grid formed by *N*_*vox*_ voxels (*N*_*vox*_ > *d*) that discretizes the (3-dimensional) volume of the brain. The *3* × *1* current density vector (source) at each voxel *u* is denoted by $\text{j}\left(u\right)=\left(j_{x}\left(u\right),j_{y}\left(u\right),j_{z}\left(u\right)\right){)}^{T}$ for *u* = 1, …, *N*_*vox*_. Here, **a**^**T**^ denotes the transpose of a vector **a**. By stacking the current vectors corresponding to all the voxels, we obtain the (stacked) current vector $\text{j}=\left({\text{j}}^{\text{T}}\left(i\right)\right){)}_{1\leq i\leq p}$, with dimension *p* = 3*N*_*vox*_. The notation *rank*(**B**)will be used for the rank of any matrix **B**.

Note that, for convenience, two equivalent notations are used for the source: as an ℝ^3^-valued field **j**(·) defined on the discrete domain *U*, and as a vector **j** ∈ ℝ^*p*^ of stacked source values over all the voxels.

The EEG “forward” or direct problem (see, e.g., Hämäläinen and Ilmoniemi, 1994; Pascual Marqui, 1999 for more details; Grech et al., 2008) is defined by the equation that determines the measured voltage **v** in terms of the source **j**:

(1) $$\begin{array}{c} \text{v}=\text{Kj}+\varepsilon , \end{array}$$

where **K** ∈ ℝ^*d*×*p*^ is known as the lead field matrix, which depends on the geometry and conductivities of the head model adopted. Here, **ε** is a measurement noise at the scalp electrodes.

After eliminating the arbitrary reference electrode through the average reference, the voltage vector **v** and the lead field **K** satisfy the following constraints (Pascual-Marqui, 2002):

(2) $$\begin{array}{c} \text{Hv}=0\in {\mathbb{R}}^{d},\\ \text{HK}=0\in {\mathbb{R}}^{d\times p}, \end{array}$$

where **H** is the *d*×*d* centering matrix

$$\begin{array}{l} \text{H}={\text{I}}_{d}-\frac{1}{d}11^{T}. \end{array}$$

Here, **I**_*d*_ denotes the *d*×*d* identity matrix, and **1** is the *d*×*1* column vector with entries 1.

Note that the constraint (2) implies that the rows of the *d*×*p* lead field matrix **K** satisfies a linear restriction, hence the rank of the matrix **K** satisfies that *rank*(**K**) ≤ *min*(*m, p*) = *m*, where

(3) $$\begin{array}{c} m=d-1. \end{array}$$

In what follows, it will be assumed the usual condition that

(4) $$\begin{array}{c} rank\left(\text{K}\right)=m. \end{array}$$

On the other hand, the EEG inverse problem consists in determining the current vector (source) **j** from the scalp electric potential vector **v** by solving the Equation (1). This algebraic linear problem has not a unique solution. A number of inverse solutions have been proposed such as the minimum norm solution (Hämäläinen and Ilmoniemi, 1994), LORETA (Pascual-Marqui et al., 1994), sLORETA (Pascual-Marqui, 2002), and eLORETA (Pascual-Marqui et al., 2011); see also Grech et al. (2008) for a review. In general, any linear inverse solutions $\hat{\text{j}}$ can be expressed in the form

(5) $$\begin{array}{c} \hat{\text{j}}=\text{Av}, \end{array}$$

where **A** ∈ ℝ^*p*×*d*^ is a specified *p*×*d* matrix, depending on the lead field **K** in a way that is determined by the particular kind of inverse solution adopted.

From and it follows that

(6) $$\begin{array}{c} \hat{\text{j}}=\text{Rj}, \end{array}$$

where the matrix **R**, given by

(7) $$\begin{array}{c} \text{R}=\text{AK}, \end{array}$$

is known as the resolution matrix. It will be assumed that the matrix **A** satisfies the ordinary condition:

(8) $$\begin{array}{c} rank\left(\text{R}\right)=rank\left(\text{A}\right)=m. \end{array}$$

Denote by **R** (*u, w*) the *3*×*3* block of the matrix **R** corresponding to two given voxels *u, w* ∈ *U*. Then, in notation of random fields, the Equation (6) can be rewritten as:

(9) $$\begin{array}{c} \hat{\text{j}}\left(u\right)=\underset{w\in U}{\sum }\text{R}\left(u,w\right)\text{j}\left(w\right). \end{array}$$

This last equation expresses the *mixing effect* in reconstructing the source by any inverse solution. Indeed, according to Equation (9), the estimate $\hat{\text{j}}\left(u\right)$ of the source field **j**(*u*) at a voxel *u* is a mixture (or weighted average) of the values of the field **j**(·) over all the voxels of the brain volume. Note that the weighting window is the row **R**(*u*, ·) of the resolution matrix **R**. Therefore, the strength of the distortion due to this mixing effect is determined by how much the resolution matrix **R** deviates from the identity matrix. In general, the condition *rank*(**R**) = *rank*(**A**) = *m* ≪ *p* (see Equation 8) implies that **R** notably deviates from the identity matrix, which usually causes remarkable distortions in reconstructing the connectivity pattern of the source. More specifically, from Equation (9) it follows that

(10) $$\text{cov}(\hat{\text{j}}(\text{u}),\hat{\text{j}}(\text{w}))\text{=}\sum_{s.r\;\in \;U}\text{R}(\text{u},\text{s})\text{cov}(\text{j}(\text{s}),\text{j}(\text{r})){\text{R}}^{T}(\text{r},\text{w}).$$

That is, the covariance $\text{cov}(\hat{\text{j}}(\text{u}),\hat{\text{j}}(\text{w}))$ between the source estimates corresponding to two voxels *u* and *w* is not the covariance cov((u),(w))jj between the true source values at the voxels *u* and *w*, but a mixture of covariance values of the true source that involves all the possible pairs of voxels in the brain volume. In section Illustrations through simulations, it will be illustrated that the connectivity distortion due to the connectivity mixing (10) can be quite drastic in many examples.

## Unmixing source estimates under segmentation

### Unmixing through arbitrary regions with completely unknown source vectors

In general, without additional assumptions, the mixing relations (9, 10), do not allow to recover the true source **j**(·) and its covariance structure from an inverse solution $\hat{\text{j}}\left(\cdot \right)$.

On the other hand, a commonly followed simplifying approach in any modality of functional brain imaging analysis is to consider a piecewise constant approximation of the image by means of a number of suitable regions, which are usually referred to as Regions of Interests (ROIs) (Poldrack, 2007; Giacometti et al., 2014; Hutchison et al., 2014). These regions used to be specified based on *a priori* (functional or/and anatomical) knowledge about the underlying neurophysiological processes in each experimental setting, or they can be just adopted as convenient simplified model for the purposes of the analysis.

The type of ROIs considered in this section is the most general case of piecewise constant source models. No additional knowledge about the source is assumed; hence, it is a practical important model for those situations in which prior information about the direction of the source vectors is not available.

In this sense, it will be elaborated in this section a general and flexible method for “unmixing” an inverse solution based on image segmentation by means of given ROIs. For this, let's specify a number *L* of ROIs *B*_1_,…, *B*_*L*_ in the brain volume *U*. A source that is constant within each of these regions has the form

(11) $$\begin{array}{c} {\text{j}}^{0}\left(u\right)=\overset{L}{\underset{l=1}{\sum }}1_{B_{l}}\left(u\right){\text{b}}_{l}, \end{array}$$

where 1_*B*_*l*__(·) denotes the indicator function of the region *B*_*l*_, and **b**_1_,…, **b**_*L*_ are *3* × *1* vectors given by

(12) $$\begin{array}{c} {\text{b}}_{l}=\frac{1}{\#B_{l}}\underset{u\in B_{l}}{\sum }\text{j}\left(u\right). \end{array}$$

That is, a source of the form (11) has constant value **b**_*l*_ over each brain region *B*_*l*_.

If (11) is regarded as a suitable model for the source **j**(·), a naive estimation of the parameters **b**_*l*_ could be given by

(13) $$b_{l}=\frac{1}{\#B_{l}}\sum_{u\in B_{l}}\hat{\text{j}}(u),$$

where $\hat{\text{j}}\left(\cdot \right)$ is the inverse solution adopted. However, this has the shortcoming that, due to the mixing effect involved in any inverse solution (see Equation 9), such estimate ${\tilde{\text{b}}}_{l}$ does not match the parameter value **b**_*l*_ even if the (11) model is exactly satisfied.

In order to overcome this difficulty, it is introduced the following alternative method: to take as estimates **b**_*l*_ of the parameters **b**_*l*_ the solution of the following system of linear equations (with respect to the unknown vectors **b**_1_,…, **b**_*L*_):

(14) $$\begin{array}{c} \frac{1}{\#B_{l}}\underset{u\in B_{l}}{\sum }\hat{\text{j}}\left(u\right)=\frac{1}{\#B_{l}}\overset{L}{\underset{s=1}{\sum }}\text{Q}\left(l,s\right){\text{b}}_{s},\left(l=1,\ldots ,L\right) \end{array}$$

where

(15) $$\begin{array}{c} \text{Q}\left(l,s\right)=\underset{u\in B_{l}}{\sum }\underset{w\in B_{s}}{\sum }\text{R}\left(u,w\right)\left(l,s=1,\ldots ,L\right). \end{array}$$

The corresponding unmixed source estimate is then given by

(16) $$\begin{array}{c} {\text{j}}^{0}\left(u\right)=\overset{L}{\underset{l=1}{\sum }}1_{B_{l}}\left(u\right){\text{b}}_{l}. \end{array}$$

Since the number *L* of ROIs is less than the number of electrodes, the condition for the solution of the Equation (14) to exist only requires that the distance between the centers of the ROIs is greater than the resolution of the inverse method used to estimate the currents at the sources. If this condition holds, the matrix Q in Equation (15) is inversible. The fulfillment of this condition for the matrix Q and the value achieved by its condition number do not depend on the data, so that they can be easily checked when specifying the ROIs.

Note that Equation (6) and (8) imply that the stacked vector $\hat{\text{j}}$ of source estimates belongs to an *m*-dimensional space. Therefore, there are at most *m* linearly independent linear functions of $\hat{\text{j}}$; and consequently, in the left hand of the system of Equation (14) only a number *L* of equations can provide non redundant information only subject to the constraint 3*L* ≤ *m*. This imposes the restriction

(17) $$\begin{array}{c} L\leq m/3 \end{array}$$

on the number *L* of ROIs to be meaningfully regarded for unmixing source estimates. Remember that we are dealing with the case in which the (constant) source **b**_*l*_ in each ROI is completely unknown, regarding both size and direction.

It can be easily shown [as a consequence of Equation (9)] that if the source model (11) holds then the Equation (14) is satisfied, hence the estimate **b**_*l*_ exactly equals the true value of the parameter **b**_*l*_ for *l* = *1,…,L*. That is, in this case the estimates **b**_*l*_ achieves an exactly unmixed version **j**^0^(*u*)of the inverse solution that matches the true source **j**^0^(*u*).

More generally, model (11) can be thought of as just an approximation to the source **j**, not exactly satisfied, by means of a conveniently specified family of ROIs. In such case, the resulting estimate is just a simplifying approximation. A bound of the resulting approximation error is given by Equations (A1, A2), in the Appendix.

Note that, according to Equation (A4), the error $\left\|\text{b}-\hat{\text{b}}\right\|$ is small either if (a) **j**^0^(·) − **j**(·) is small [i.e., if model (11) is exact enough]; or (b) the resolution matrix **R** is close to the identity matrix [in which case **R**(*u, w*) is near zero for any two distinct voxels *u* and *w*]. These descriptive statements have the precise quantitative meaning conferred by the vector norms involved in the Equation (A4).

Obviously, the piecewise constant model (11) for a source field tends to be a better approximation as the number of regions increases. This suggests to set the number *L* of ROIs as large as the restriction (17) allows, and to increase the number of EEG recording electrodes as much as possible to have a larger upper bound *m*/3.

Also, it is worth of emphasizing that the approach for unmixing just introduced here offers comprehensive generality and flexibility regarding the choice of the ROIs *B*_1_,…, *B*_*L*_. These can be arbitrary regions selected for a specific experimental setting by the researcher. Some distinguished, commonly useful types of ROIs are the following:

**(S1)** *Poin*t brain regions.

**(S2)** *Surface* regions (e.g., a segmentation of the brain cortex surface).

**(S3)** *Three-dimensional* (3D) brain regions.

**(S4)** A family of regions that includes different types (**S1**)–(**S3**).

Moreover, the definition of the unmixing operator Q in Equations (15) and (22) indicates that it depends on the inverse solution method used (be this any of the Loreta family, the Minimum Norm family or other) only through its Resolution matrix R (Equation 7). Therefore, the only factor of the inverse solution that affects our unmixing procedure is its specific resolution. This determines the minimum possible distance between the sources to be unmixed that is allowed to keep model identifiability (i.e., existence of the inverse of **Q**), since obviously two sources that share a common region in the resolution space of the inverse solution cannot be distinguished. To further clarify this statement, consider, for example, sLoreta and Minimum Norm estimate (MNE). MNE has slightly better resolution than sLoreta if the sources are estimated at the surface of the cortex. However, when estimating deep sources, MNE may lead to higher localization errors and ghost solutions, while sLoreta maintains its property of zero localization error (Grech et al., 2008; Jatoi et al., 2014). In that sense, it may be a good choice to select MNE (although MNE does not guarantee zero localization error even at the surface) when working with superficial lead fields, while it would be the appropriate selection to use sLoreta when dealing with volumetric Leadfields. In both cases, the unmixing procedure is only affected by the distance of the sources to be unmixed, which should not be smaller than the resolution of the method.

### Unmixing through regions on the cortex surface with known source directions

The maximum number of regions that satisfies the constraint (i.e., *m/*3) is quite small if the number *d* of scalp electrodes is not large (see Equation 3). For example, *m* = 18 in the 1,020 EEG recording system, hence *m/*3 = 6.

A way to overcome this limitation is to impose additional simplifying assumptions on the (approximate) source model. In particular, in this section it will be assumed the following source model instead of Equation (11): at each voxel *u* of a given ROIs *B*_*l*_, the source has a known direction **d**(*u*) (with ||**d**(*u*)|| = 1) and a common intensity *a*_*l*_; that is, Equation is replaced by

(18) $$\begin{array}{c} {\text{j}}^{0}\left(u\right)=\overset{L}{\underset{l=1}{\sum }}1_{B_{l}}\left(u\right)\text{d}\left(u\right)a_{l}, \end{array}$$

where *a*_1_,…, *a*_*L*_ are scalar values defined by

(19) $$\begin{array}{c} a_{l}=\frac{1}{\#B_{l}}\underset{u\in B_{l}}{\sum }{\text{d}}^{T}\left(u\right)\text{j}\left(u\right). \end{array}$$

Note that the projection of **j**^0^ (*u*) onto the known direction **d**(*u*) results in the scalar field

(20) $$\begin{array}{c} X^{0}\left(u\right)=\overset{L}{\underset{l=1}{\sum }}1_{B_{l}}\left(u\right)a_{l}. \end{array}$$

In principle, this type of approximation can be applied for brain segmentations into arbitrary ROIs *B*_1_,…, *B*_*L*_. For easier interpretation in the simulated examples shown below, in this section we will focus in the case of regions of the cortex surface. Consequently, the inverse solution $\hat{\text{j}}$ will be restricted to the cortex surface, i.e., the matrix **A** and the resolution matrix **R** in Equations (5) and (7), are computed based on a reduced lead field $\tilde{\text{K}}$ obtained by retaining only the columns of the lead field **K** corresponding to voxels on the cortex surface.

The unknown parameters in Equations (18 – 20) are the scalar values *a*_1_,…, *a*_*L*_. They will be estimated by the solution *â*_1_, …, *â*_*L*_ of the following system of *L* linear equations:

(21) $$\begin{array}{c} \frac{1}{\#B_{l}}\underset{u\in B_{l}}{\sum }{\text{d}}^{T}\left(u\right)\hat{\text{j}}\left(u\right)=\frac{1}{\#B_{l}}\overset{L}{\underset{s=1}{\sum }}q_{ls}a_{s},\left(l=1,\ldots ,L\right) \end{array}$$

where

(22) $$\begin{array}{c} q_{ls}=\underset{u\in B_{l}}{\sum }\underset{w\in B_{s}}{\sum }{\text{d}}^{T}\left(u\right)\text{R}\left(u,w\right)\text{d}\left(w\right)\left(l,s=1,\ldots ,L\right). \end{array}$$

The resulting unmixed source estimate is given by

(23) $$\begin{array}{c} {\hat{\text{j}}}^{0}\left(u\right)=\overset{L}{\underset{l=1}{\sum }}1_{B_{l}}\left(u\right)\text{d}\left(u\right){\hat{\text{a}}}_{l}, \end{array}$$

and its corresponding projection onto the known direction field **d**(.) is the scalar field

(24) $$\begin{array}{c} {\hat{X}}^{0}\left(u\right)=\overset{L}{\underset{l=1}{\sum }}1_{B_{l}}\left(u\right){\hat{\text{a}}}_{l}. \end{array}$$

Since these equations involve only *L* linear functions of the inverse solution $\hat{\text{j}}$, it is admissible to adopt any number *L* of regions with the constraint

(25) $$\begin{array}{c} L\leq m \end{array}$$

Note that this constraint contrasts with the stronger one *L* ≤ *m/3* that was required in the previous section.

Similarly to results in the previous section, it can be straightforwardly shown that this method provides estimates ${\hat{\text{j}}}^{0}\left(\cdot \right)$ and ${\hat{X}}^{0}\left(\cdot \right)$ that, in contrast to the inverse solution $\hat{\text{j}}\left(\cdot \right)$, exactly recover the true source **j**(·) when the model (18) is satisfied (i.e., when **j**(·) = **j**^0^(·)). In other words, this estimation method achieves exact unmixing of the inverse solution under this condition. Otherwise, it leads to an approximate unmixing whose error depends both on the satisfiability of the model (18) and the resolution **R**, analogously as was shown in the previous section.

### Unmixing inverse solutions vs. directly fitting the model to the data

From certain point of view, the unmixing procedure consists in correcting a mistake introduced by the inverse solution method used to obtain the currents at the sources. This mistake is unavoidably caused by the ill-posed mathematical nature of the inverse problem, where the number of unknowns is much bigger than the number of observables. This fact might suggest considering the convenience of using a simpler approach in which the estimates at the sources are obtained via direct estimation of the model from the data, without requiring a previous step of distributed inverse solution as sLORETA. In principle, this approach may avoid solving an ill-pose problem by restricting the solution to a small number of unknowns, which is less or equal to the number of observations.

More specifically, this approach can be modeled as follows:

(26) $$j=\sum_{l=1}^{L}\underset{l}{\alpha }1_{A_{l}}=M\alpha ,where\underset{l}{\alpha }\in \mathbb{R}\;and\;1\in {\mathbb{R}}^{N_{vox}}$$

(27) $$\begin{array}{c} 1_{A_{l}}^{u}=\left\{\begin{array}{c} 1\;if\;u\in A_{l}\\ 0\;if\;u\notin A_{l} \end{array}\right\} \end{array}$$

(28) $$\begin{array}{c} M=\left[1_{A_{1}}\ldots 1_{A_{L}}\right],M\in {\mathbb{R}}^{N_{vox}xL} \end{array}$$

(29) $$\begin{array}{c} \alpha =\left(\begin{array}{c} \alpha_{1}\\ \ldots \\ \alpha_{L} \end{array}\right) \end{array}$$

Under this formulation, the Equation (1) can be rewritten as:

(30) $$\begin{array}{c} \text{v}=\text{Kj}+\varepsilon =\text{K}M\alpha +\varepsilon . \end{array}$$

The minimum least square solution of Equation (30) is given by:

(31) $$\overset{\wedge }{\alpha }={\left(M^{T}K^{T}K\;M\right)}^{-1}M^{T}K^{T}V$$

The corresponding estimate of current at the sources is obtained by:

(32) $$\hat{j}=\sum_{l=1}^{L}{\hat{\alpha }}_{l}1_{Al}=M\hat{\alpha }$$

Equations (8), (17), and (25) state that L is always less than the rank of K. This condition guarantees the existence of the inverse in Equation (31) and therefore the solution of Equation (31) always exist and it is unique. The difference with the inverse solution estimates is that in this case, only a small number of sources (L) are estimated instead of the whole set of sources in the gray matter, as it is the case with the inverse methods. This should not be considered a limitation, since anyway, after estimating the inverse solution, only L sources can be unmixed.

Through simulations in the next section this direct fitting procedure will be tested against the unmixing of the distributed inverse solutions procedure.

## Illustrations through simulations

**Figure 1:**
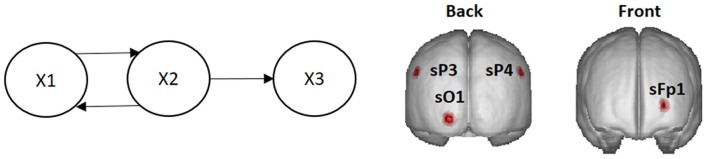
**Left**: System diagram. The past of X1 influences the present of X2, and vice versa. The past of X2 influences the present of X3. X3 does not influence any variable. **Right**: Voxels in the cortex used as locations of point sources in the simulations (named according to the 10–20 EEG recording system).

Let X1, X2, and X3 be the (time-varying) state variables of a three-variate system with the dynamic connectivity pattern shown in Figure 1.

The linear interactions between the variables are set according to the following multivariate auto-regressive (MAR) order 2 model:

(33) $$\begin{array}{c} \text{X}\left(t\right)=\text{A}\left(1\right)\text{X}\left(t-1\right)+\text{A}\left(2\right)\text{X}\left(t-2\right)+\text{e}\left(t\right), \end{array}$$

where

(34) $$\begin{array}{c} \text{X}\left(t\right)={\left(X_{1}\left(t\right),X_{2}\left(t\right),X_{3}\left(t\right)\right)}^{T},\left(t=1,2,\ldots \right); \end{array}$$

(35) $$\begin{array}{c} \text{A}\left(1\right)=\left(\begin{array}{ccc} 1.5 & -0.25 & 0\\ -0.25 & 1.8 & 0\\ 0 & 0.5 & 1.3 \end{array}\right),\\ \text{A}\left(2\right)=\left(\begin{array}{ccc} -0.95 & 0 & 0\\ 0 & -0.96 & 0\\ 0 & 0 & -0.95 \end{array}\right), \end{array}$$

and the time series **e**(*t*) is a white noise, with zero mean and variance 1, i.e., with covariance matrix:

(36) $$\begin{array}{c} V\left(\text{e}\right)=\text{I}. \end{array}$$

To assess the causal connectivity patterns between the variables of the system, a direct and directed measure of causal influence in the frequency domain, named “Isolated Effective Coherence” (iCoh) (Pascual-Marqui et al., 2014), is used here. For each pair of system variables *X*_i_ and *X*_j_, the iCoh index from *X*_i_ toward *X*_j_ is defined as the partial coherence between them (at each frequency) under a multivariate autoregressive model obtained from the original one by setting all irrelevant associations (autorregressive coefficients) to zero, other than those corresponding to the particular direct and directional influence of interest from *X*_i_ toward *X*_j_. Unlike the correlation, iCoh is not symmetric and can distinguish between direct and indirect causal flow of information, so capturing the patterns of directed influences between the variables. For more details and examples of the iCoh connectivity measure (see Pascual-Marqui et al., 2014).

**Figure 2:**
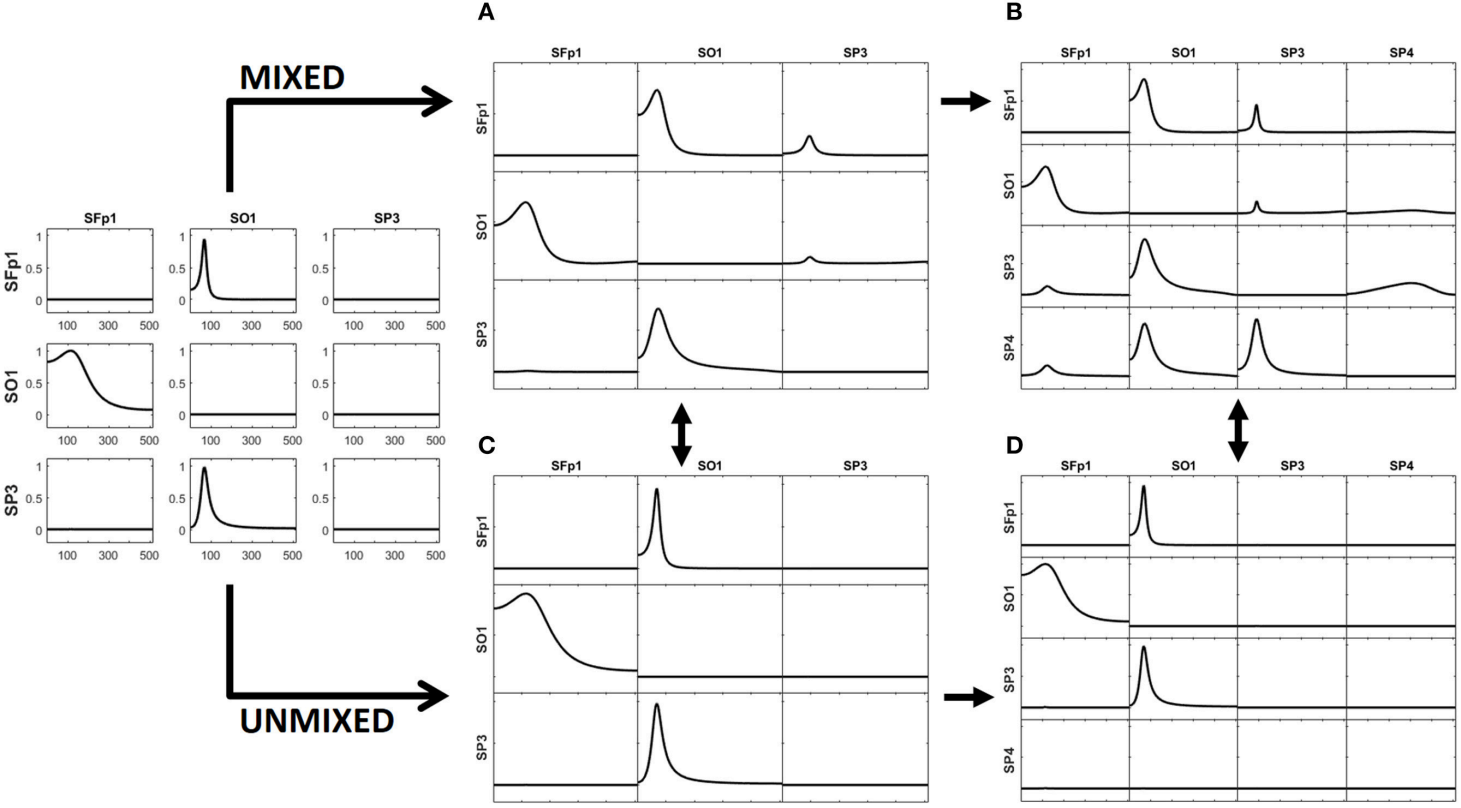
To the left, the iCoh results of connectivity between the original variables used for the simulation. Panel **(A)** shows the iCoh measures of connectivity based on the (mixed) current estimates by sLoreta at the point sources considered in the simulation. Panel **(B)** shows that spurious patterns appear when adding in the analysis new voxels that were not originally considered in the simulation of the source. Panels **(C,D)** show the exact reconstruction of the connectivity patterns achieves by applying the unmixing procedure. Panel **(B)** for the voxels included in the simulation of the source; panel **(D)** when adding new voxels not included in the simulation. In both cases, the reconstruction of the original patterns is exact.

The left panel in Figure 2 illustrates the real pattern of connectivity that were simulated [according to (33)-(36)] between X1, X2 and X3, recovered by iCoh.

### Point ROI-based unmixing with exact source models

Different simulations presented here illustrate the mixing effects produced by EEG inverse methods, as were discussed in section Mixing effects in source estimation. The signals X1, X2, and X3 were assigned to the nearest voxels in the cortex to the electrodes Fp1, O1 and P3 of the 10–20 EEG recording system (panel to the right in Figure 1). Furthermore, an additional white noise with zero mean and 0.1 standard deviation was added at all voxels of the brain.

A superficial three concentric spheres lead field (Hoke et al., 1990; Riera and Fuentes, 1998; Bosch-Bayard et al., 2001), for a grid defined over the surface of the cortex of the Montreal Neurological Institute (MNI) (Evans et al., 1993), was used to solve the forward EEG problem and generating the voltage at the 19 electrodes of the 10–20 System.

The method described in section Unmixing through regions on the cortex surface with known source directions will be used for unmixing, taking point ROIs on the cortex surface. For this, the lead field was projected to the normal direction to the surface for all voxels. This allows the estimation of 18 independent point sources (see Equation 37).

The generated voltage was used to go back to the source space by solving the EEG inverse problem with sLoreta on the cortex surface. In this way, an estimate of the current density at any voxel (point source) on the cortex surface was obtained. iCoh was used to infer the connectivity between point sources based on their reconstructed signals by means of sLoreta. Figure 2A shows the connectivity patterns recovered by iCoh from the sLoreta-reconstructed signals at SFp1, SO1, and SP3. The iCoh patterns are recovered with a high degree of similarity (compare with the patterns shown to the left in Figure 2).

However, if one additional voxel is added in the connectivity analysis, as SP4 in Figure 2B, many spurious patterns of connectivity appear between the voxels. The shortcomings become aggravated as more voxels are added to the analysis. This effect is due to the mixing effect of the EEG inverse method, sLoreta in this case. The results from Figures 2A,B may suggest that, in order to be able of recovering the correct patterns of connectivity with the inverse solutions, one must know the exact set of variables sending and receiving information in the system, a non-feasible supposition in most experimental settings.

The unmixing procedure described in section Unmixing through regions on the cortex surface with known source directions for regions on the cortex surface solves this problem, using point ROIs brain regions [i.e., regions described as type (**S1)** in section Unmixing through arbitrary regions with completely unknown source vectors]. The unmixed signals recover the exact patterns of connectivity of the point sources included in the simulation. Indeed, Figure 2C shows the exact reconstruction of the connectivity patterns for the real system that is obtained after unmixing. Moreover, adding new point ROIs (or variables) in the analysis of the unmixed signals neither affects the patterns of connectivity between the original variables nor introduce spurious information flow with the additional variables, as is shown in Figure 2D.

### Simulations of unmixing inverse solutions vs. directly fitting the model to the data

**Figure 3:**
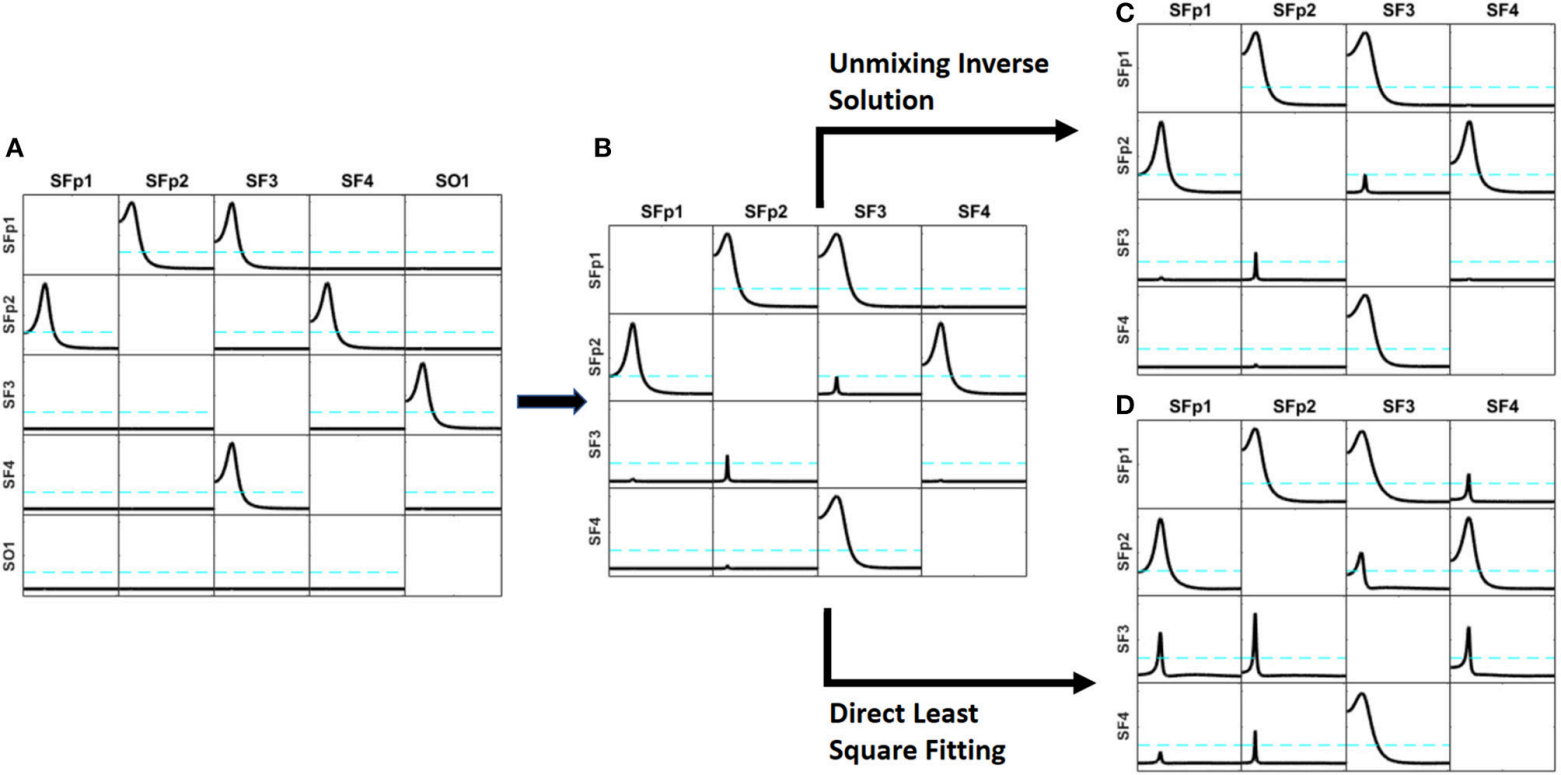
Comparison of the results obtained by the unmixing procedure after the distributed inverse solution vs. the direct solution of the problem by Least Square solution. Panel **(A)** shows the exact simulation used. Panel **(B)** shows the reconstruction of the patterns of influence by iCoh when one of the simulated signals (sO1) is ignored; observe that very small artifacts appear. Panel **(C)** shows the iCoh results using the signals reconstructed at the sources after solving the inverse problem and applying the unmixing procedure ignoring sO1; note that it almost the same as Panel **(B)**, no better result can be expected. Panel **(D)** shows the iCoh reconstruction of influences using the signals obtained by direct estimation of the model by Least Square ignoring sO1; observe that many spurious patterns appear where the influence should have been zero, although the real patterns between the included regions are well recovered.

To test whether the approach introduced in section Unmixing inverse solutions vs. directly fitting the model to the data may produce better results than the unmixing procedure, we performed the following simulation:

(a) Select the 5 nearest sources to Fp1, Fp2, F3, F4, and O1 (which will be denoted by sFp1, sFp2, sF3, sF4, and sO1).

(b) Using the methodology explained in section Illustrations through simulations (Equations 33–36), generate time series for each of the 5 sources. The patterns of influence among the variables is shown in Figure 3A: Fp1 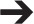 sFp2; sFp2 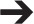 sFp1; sF3 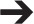 sFp1; sF3 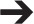 sF4; sF4 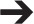 sFp2 and sO1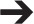 sF3.

Using the time series set to the mentioned sources, the forward problem was solved as described in section Point ROI-based unmixing with exact source models to produce measurements for the voltage at the 19 electrodes of the 10–20 system. Note that these simulation settings are similar to the ones used to validate methods in Baccalá and Sameshima (2001); Baccala et al. (2007) and Pascual-Marqui et al. (2014).

On the basis of the voltage signals, two procedures were used to reconstruct the signals back at the same sources where the signals had been simulated (sFp1, sFp2, sF3, sF4, and sO1): (a) solving the inverse solution with sLoreta and then applying the unmixing procedure as explained in section Unmixing through regions on the cortex surface with known source directions; and (b) directly estimating the current at the selected sources using the methodology explained in section Unmixing inverse solutions vs. directly fitting the model to the data. When solving the inverse problem and applying the unmixing method, the procedure produces estimates of the current for all sources in the grid, while with the direct procedure, current estimates are obtained only for the 5 selected sources. After that, the iCoh technique was applied to reconstruct the patterns of influence at the sources, using the reconstructed time series.

When unmixing and direct solutions were performed using the same 5 selected sources, both procedures reconstructed the exact patterns of causality as generated. They are not shown because it was an obvious result.

However, the results are quite different when, instead of using the 5 selected sources, we ignore one of them (sO1 in this case) and reconstruct the patterns of influence using only 4 of the 5 sources: sFp1, sFp2, sF3, and sF4. When ignoring one of the variables and calculating iCoh with the rest of them, the iCoh technique cannot reconstruct the exact patterns of causality since important information about the multivariate system is omitted; namely, some of its variables. However, as shown in Figure 3B, the artifacts recovered by iCoh from this inexact model are minor. Indeed, Figure 3B, shows almost exact reconstruction of the patterns of influence among the original system, though the time series set at sO1 was disregarded in computing iCoh measures. Only two small artifacts appear—from sFp2 to sF3, and from sF3 to sFp2. The rest of the patterns remain as they were simulated.

Figure 3C shows the results obtained by solving the inverse problem with sLoreta, selecting the time series reconstructed at the 4 sources (sFp1, sFp2, sF3, and sF4) and applying the iCoh technique to them, after the unmixing procedure. The results are almost exactly the same ones obtained for the original times series in Figure 3B, i.e., the unmixing procedure results are as good as the best it can be expected in such situation.

However, the results from calculating iCoh with the signals estimated by the direct procedure results in many artifacts in the influence patterns, not present in the original simulation, as shown in Figure 3D. Tough the real patterns of influence between are correctly reproduced, additional spurious patterns of influence are present in almost all the pairs of sources. In some of them, like sF3 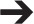 sFp2, the spurious pattern involves more than one frequency.

Similar results are obtained in a wide variety of simulated scenarios. In general, the unmixing procedure shows to be more robust than direct fitting concerning deviations of the adopted model from the true source distribution.

The greater robustness of the unmixing approach regarding misspecification of the source model is theoretically understandable. Distributed inverse solutions (as LORETA) have low resolution, but they have at least some amount of resolution. Therefore, including a distributed inverse solution as a previous step provides some degree of source localization. Consequently, the confounding effects due to sources not included in the adopted model are diminished by means of the distributed inverse solution step involved in computing the unmixing solution.

#### Effect of additive measurement noise in the recovery of connectivity patterns

In this section, the effect of the additive measurement noise **ε** [specified in the Equation (1)] is considered in more detail within the context of unmixing for recovering source connectivity patterns. This noise appears during the recording of the EEG due to technical reasons and has nothing to do with the subject's brain activity.

To test the effect of this noise, a simulation like the one used in section Simulations of Unmixing inverse solutions vs. directly fitting the model to the data is repeated here. The only difference is that, after the voltage at the electrodes is obtained by solving the forward problem, now in this case we also add a white Gaussian noise to the voltage before reconstructing the signal back at the sources by means of sLoreta. This is a second type of noise different from the noise added at the time signals at the sources during the simulation.

From this noise-corrupted voltage we reconstruct the patterns of influence at the sources in the same way we did in section Simulations of Unmixing inverse solutions vs. directly fitting the model to the data (again omitting sO1 in the iCoh technique calculation), by means of the two procedures: (a) unmixing after sLoreta; and (b) directly estimating the current at the selected sources using the methodology explained in section Unmixing inverse solutions vs. directly fitting the model to the data.

**Figure 4:**
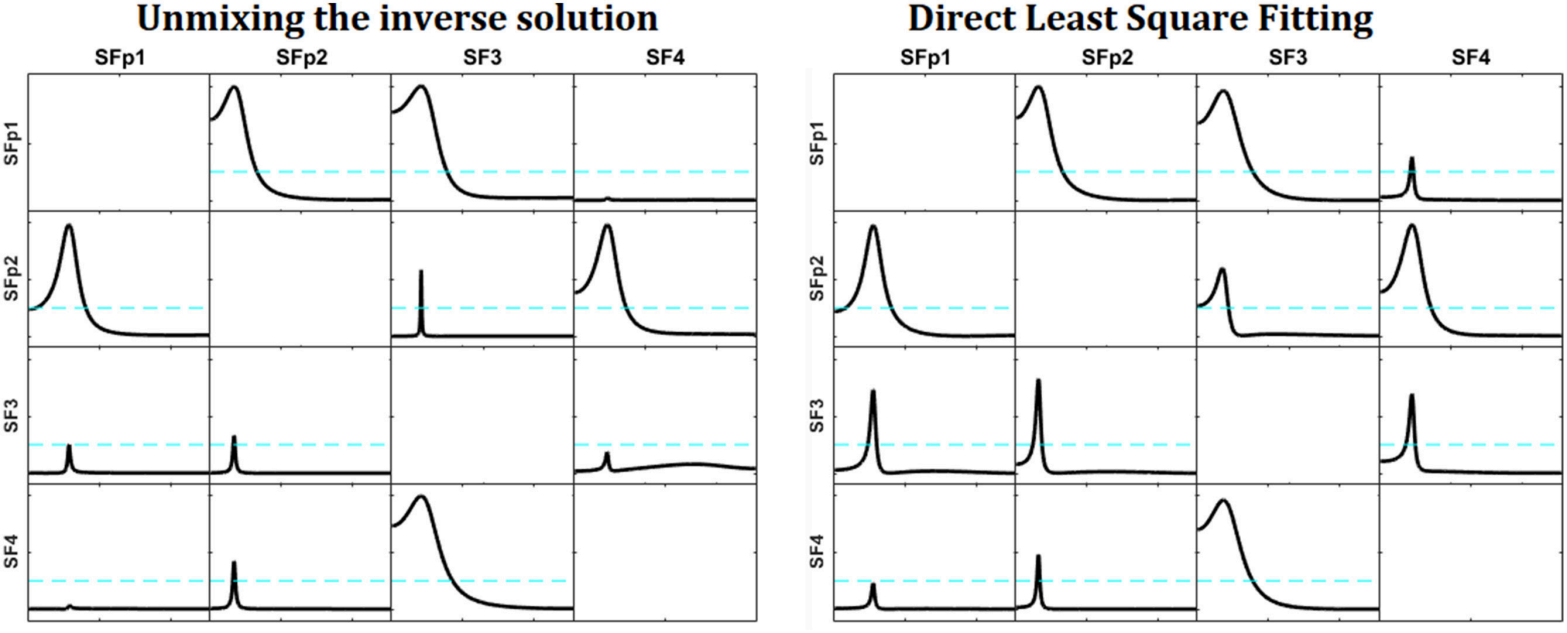
Reconstruction of the patterns of influence for the system simulated in Figure 3 with measurement noise added. At the left, the reconstruction obtained by the unmixing procedure and sLoreta. To the right, the reconstruction obtained by the direct fitting of the model. Note that the reconstruction of the unmixing procedure is less affected by the noise, and very similar to the one obtained in Figure 3, where the measurement noise was not added.

Figure 4 shows the reconstructed patterns of influence for the two methods. At the left, the reconstruction of the causality patterns by the unmixing algorithm applied to the inverse solutions; the right panel shows the reconstruction of the patterns using the direct fitting of the model. The patterns obtained by the unmixing procedure are less affected by the noise than the ones obtained by the direct fitting. Compare them with those in Figure 3.

We repeated this simulation for different amounts of noise [decreasing the signal to noise ratio (SNR)] and increasing the number of sources to be unmixed. It is worth to say, that the direct fitting is more resistant to the decrease of the SNR than the sLoreta estimates. Both procedures are affected by the increment of the measurement noise (as expected), but the sLoreta estimates are affected in a more severe way than the direct fitting. Also, when increasing the number of sources to be unmixed, as we approach to the maximum number of sources possible to be unmixed (the number of electrodes minus 1), the stability of the sLoreta procedure is affected in a more severe way than the direct fitting.

At this point, we consider necessary to give here two additional comments: in the literature of brain connectivity it is not frequent that researchers include the analysis of more than 10 brain regions; and second, although the direct fitting is less affected than sLoreta when the noise increases, the gain is not clear since any of the two methods is able to correctly reproduce the connectivity patterns. In this sense, it is worth to mention that the problems mentioned above are not inherent limitations of the unmixing procedure itself but rather than of the sLoreta method. There exist other types of distributed inverse methods which are less sensitive to the noise than sLoreta; for example: eLoreta. While the inverse method is more resistant to the noise, the unmixing procedure will perform with more stability.

Thus, by one side, the unmixing procedure inherits virtues of the distributed inverse solution used, such as some amount of model-free resolution that leads to robustness regarding model misspecification, the more the better be the resolution (as discussed in section Simulations of Unmixing inverse solutions vs. directly fitting the model to the data above). On the other hand, also may inherits some drawbacks regarding noise-sensitivity that are specific to the distributed inverse solution adopted, as discussed in the present section. Selecting a suitable trade-off between resolution and noise-sensitivity in each concrete situation is a general problem dealt with in the research about distributed inverse solutions. A remarkable advantage of the flexibility of the introduced unmixing procedure is that it allows incorporating any proposal from this research literature.

### Performance of the unmixing procedure with different inverse methods

In the last paragraph of section Unmixing through arbitrary regions with completely unknown source vectors, the conditions for the existence and inversibility of the unmixing operator Q and for the possibility of unmixing two specific sources are analyzed. In this section we illustrate this fact through examples. For convenience, we use the same example of section Simulations of Unmixing inverse solutions vs. directly fitting the model to the data and compare the performance of two inverse methods: Minimum Norm Estimates (MNE) and sLoreta.

**Figure 5:**
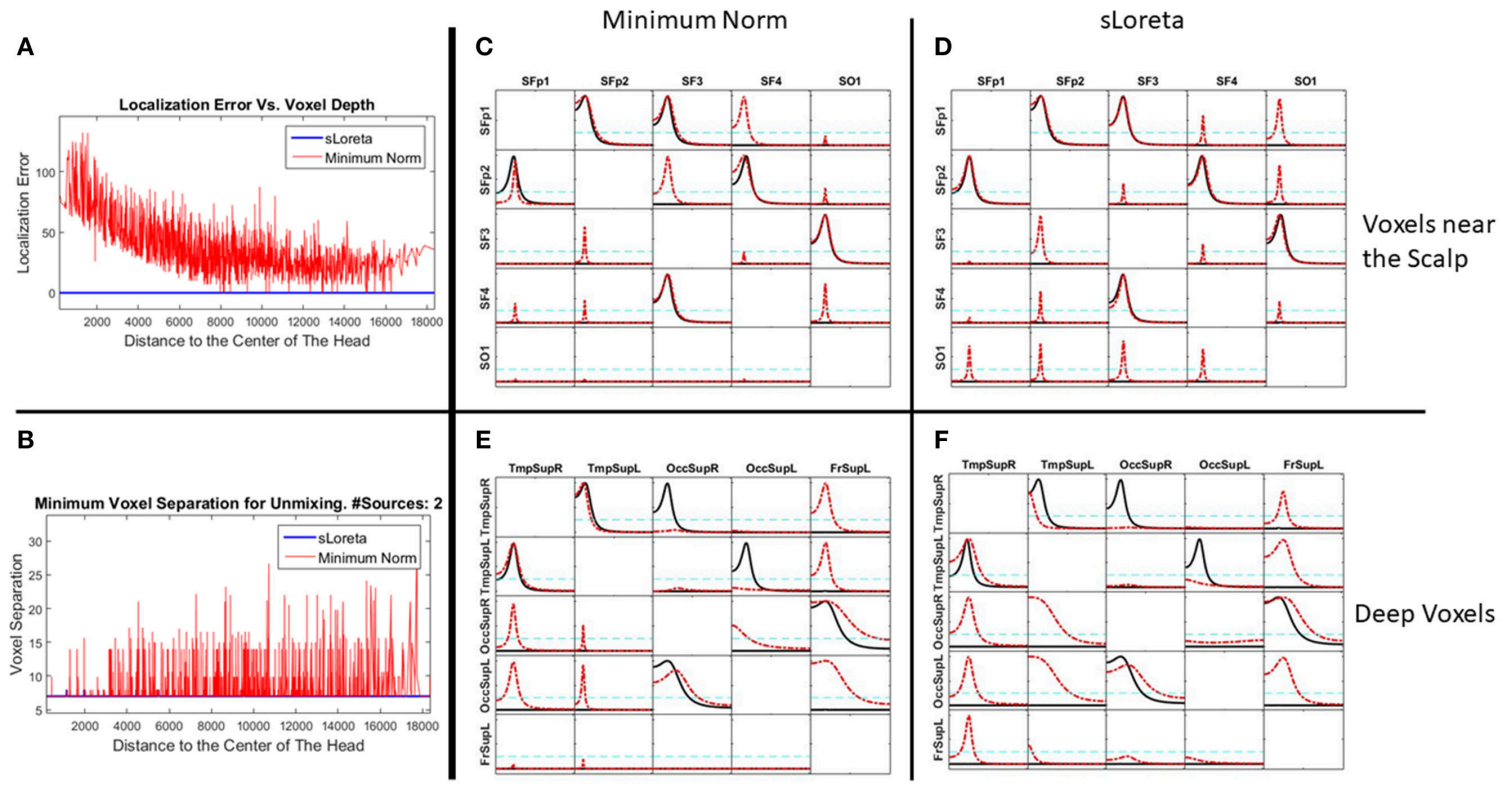
Performance of the unmixing procedure for the Minimum Norm and sLoreta, for voxels at different depth in the brain. Panels **(A,B)** show a comparison of MNE and sLoreta in terms of localization error and resolution matrix. Panels **(C–F)** show the connectivity patterns for a set of voxels near the scalp **(C,D)** and a set of voxels deeper in the brain **(E,F)** for MNE **(C,E)** and sLoreta **(D,F)**. The reconstruction of the causality after unmixing is perfect in all situations.

Figure 5 is divided in 6 panels. Panel A and B illustrate some properties of the two inverse methods under comparison. First, panel A illustrate, just as a remind, the known localization error for the two methods. The X axis contains all the voxels considered in the grid (3244) sorted by their distance to the center of the head (HC). A smaller value of X indicates proximity to HC, while the voxels near the scalp have the highest distance to the HC. The blue solid line in zero along the X axis indicates the sLoreta localization error, which is known to be zero, as a property of sLoreta. For the minimum norm (line in red), voxels near the HC (deeper voxels) have bigger localization error, which diminishes while the distance to the center increases, i.e., voxels near the scalp.

Figure 5B shows the minimum distance between two sources necessary for the unmixing procedure (inversibility of Q). Again, the X axis represents the voxels according to their distance to the HC and the Y axis is the distance between the two voxels. The blue line shows that with sLoreta, almost all the voxels (except 4) can be unmixed even with the nearest neighbor. However, for the MNE (red line) many voxels need a bigger separation for the unmixing procedure. There is not a clear tendency between the depth of the voxels and the minimum distance for unmixing, although it appears that deeper voxels have smaller distances, i.e., they can be unmixed.

In Figures 5C,D, the five voxels nearest to the S1020 channels Fp1, Fp2, F3, F4, and O1 are selected as targets for the simulation. The five signals of the example in section Simulations of Unmixing inverse solutions vs. directly fitting the model to the data are set to them. Then, the voltage at the scalp is generated by solving the forward problem and again, the signal at the sources are estimated by means of MNE and sLoreta. The patterns of causality are recovered by calculating the iCoh between the signals, before and after the unmixing procedure.

Figures 5C,D shows the performance of MNE and sLoreta respectively. In both, the red dashed lines show the causality patterns estimated by iCoh with the signals before unmixing and the solid black lines show the recovery of the causality with the unmixed signals.

Figures 5E,F show the results for a similar simulation but selecting the target voxels in deeper regions of the brain: two at the Temporal Superior (Right and Left), two at the Occipital Superior (Right and Left) and one at the Left Frontal Superior region.

Figures 5C–F show that the causality patterns of the voxels before the unmixing procedure are very affected by the mixing effect both for sLoreta and MNE. However, after unmixing, the recovery of the causality patterns in the four situations is perfect. This result is important since it confirms the statements written in section Unmixing through arbitrary regions with completely unknown source vectors that the unmixing procedure does not depend neither on the inverse method, nor on the localization error or the depth of the sources under consideration. It is also worth to note that MNE performs well for connectivity matters after applying the unmixing procedure, despite the worse localization error and resolution matrix than sLoreta.

These results are in consonance with Hedrich et al. (2017) who made a comparison of performance of different inverse methods, including MNE and sLoreta. Our results show the same localization error for both methods, although here we reported it in terms of the voxel depth. Hedrich et al. (2017) also reported a similar amount of leakage for MNE and sLoreta, which is in concordance with our results, as it can be observed in Figure 5. Additionally, they showed that the spatial dispersion of the Point Spread Function (PSF) of sLoreta is bigger than the one for the MNE. This looks to be consistent with Figure 5, where sLoreta shows a more widespread mixing effect than MNE.

### Point ROI-based unmixing with exact source models of systems with more variables

**Figure 6:**
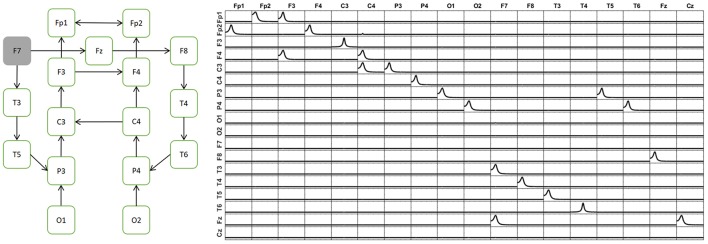
Simulation of a system with 17 variables. The diagram in the **left panel** shows the map of connectivity patterns simulated among the sources. The **right panel** shows the reconstruction of the connectivity patterns among the signals, using iCoh. Note that the reconstruction is exact.

To illustrate that the unmixing procedure works perfectly even in the boundaries of the maximum admissible number of point sources as ROIs (18 in this case), a more complex system is simulated, involving 17 variables, with very complex patterns of connections between them (see Figure 6, left panel). The same kind of MAR system previously simulated for X1, X2 and X3 have been extended here between all the 17 variables to construct this complex system. For clarity in further use, they are named as the corresponding electrodes of the 10–20 recording EEG. The arrows indicate the direction of the influence. There is a bidirectional flow of information between Fp1 and Fp2. In the rest of the voxels, the information flow goes in only one direction. The right panel of Figure 6 shows the exact patterns of causality of the simulated system obtained by iCoh.

**Figure 7:**
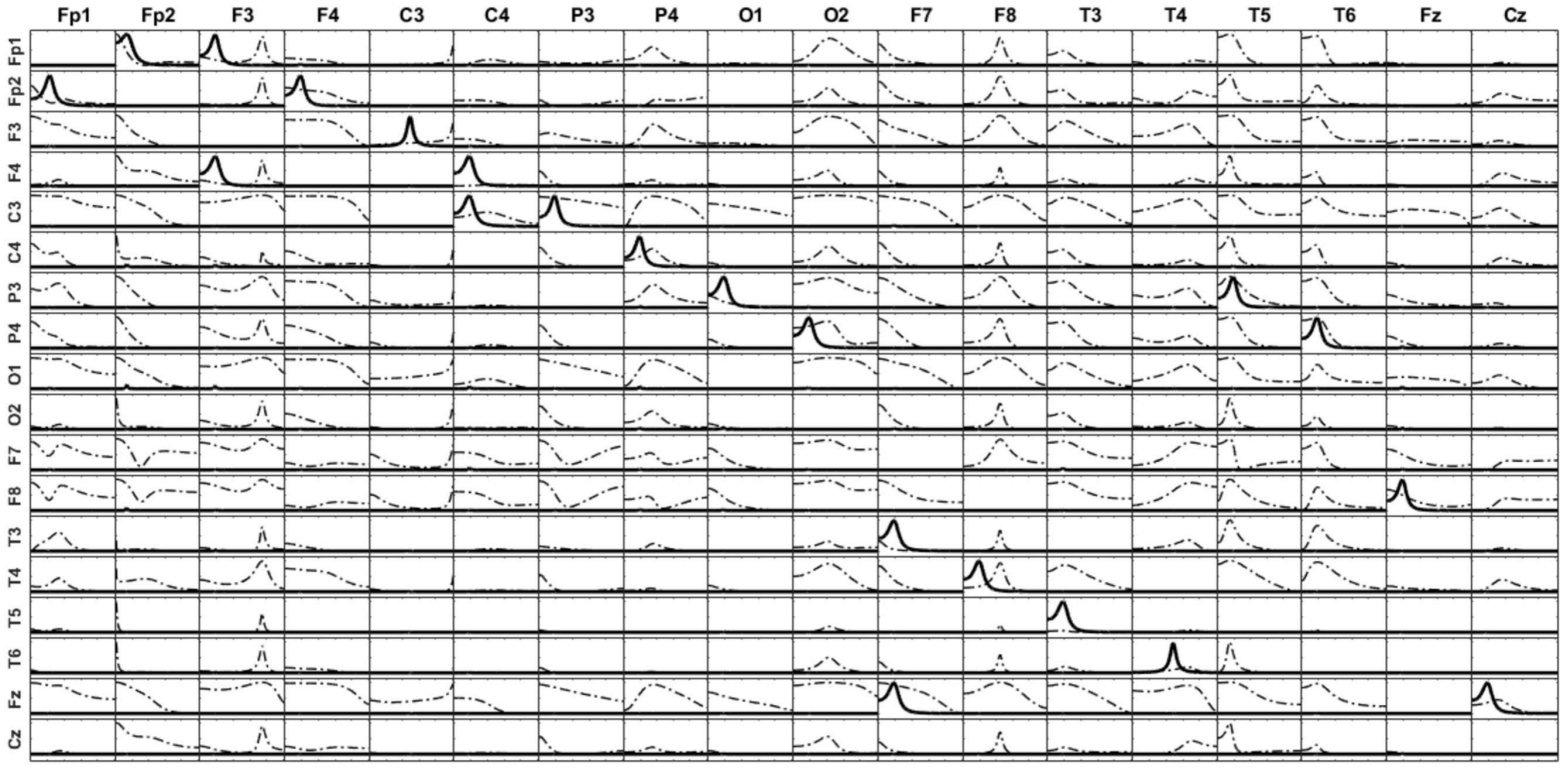
iCoh measures of the 17 sources of the reconstructed system. Solid thick lines after unmixing the sLoreta reconstruction; dashed lines before unmixing.

The signal of each variable of this system is then set as a point source at the voxel of the cortex closest to the electrode corresponding to its given name. From the source composed by such set of point sources, the voltage at the 10–20 EEG electrodes are obtained by solving the EEG forward problem. Then, the current densities are estimated at the sources by means of sLoreta. The iCoh patterns of connectivity based on the these (mixed) reconstructed signals at the 17 voxels are shown in Figure 7 in dashed lines. The mixing effect of the inverse solution produces a big number of spurious patterns of connections between the signals estimated at the sources, as is shown by the dashed lines in Figure 7. After applying the unmixing procedure, the iCoh connectivity patterns between the point sources are recovered exactly as with the original simulated signals. They are shown in Figure 7 in solid lines; compare them with the right panel of Figure 6.

### Point ROI-based unmixing from inexact source models

Now, to illustrate the effect of estimating the causal inferences with an inexact source model, we provide simulation results in three additional situations. In all of them, the simulated (true) source is the one represented to the left in Figure 6.

**Figure 8:**
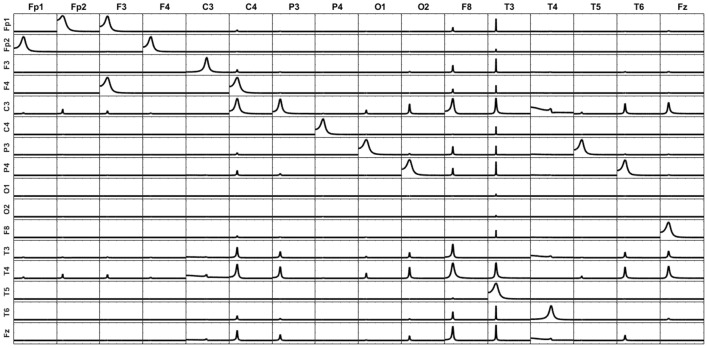
iCoh for 16-point sources reconstructed by unmixing without including one of the original point sources (F7) in the analysis. F7 is considered neither in the unmixing procedure nor in the iCoh calculation. The unexplained causality due to F7 appears as artifacts between the rest of the variables.

First, let's remove one voxel of a point source from the source model (F7 in this case). The unmixing procedure is then carried out over the remaining 16 voxels instead of the 17 voxels involved in the simulated system. The iCoh measure is computed based on the unmixed signals at such 16 voxels. Figure 8 illustrates the iCoh results in this situation. Although the original patterns of causality between the 16 voxels are kept intact, some additional spurious patterns of connectivity appear between them. The reason for this result is that the unmixing procedure did not include F7, and therefore there is still some mixing effect between the reconstructed signals.

**Figure 9:**
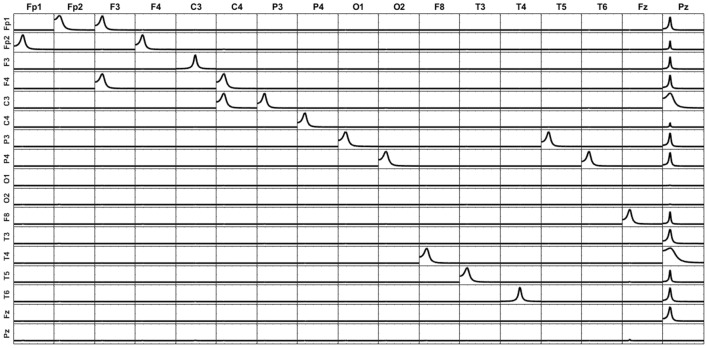
iCOH of the 17-point sources obtained by removing F7 and adding a new voxel (Pz) which was not in the original system. All the unexplained causality appears as artifacts in Pz.

The second situation consists in replacing in the unmixing procedure one-point source involved in the simulated system (F7 as in the previous simulation) by another point source which did not participate in the original system, and so does not contain relevant connection patterns (Pz). Figure 9 shows that in this case all the artifacts are explained by the additional variable, while the connectivity patterns between the 16 original variables included in the analysis remain intact. This effect has been previously described by Wong and Ozaki (2007) for the case when the innovations from the estimation of the multivariate AR model is not diagonal (not white innovations), meaning that some variance of the system is still not explained by the model. In this case, they propose to add an external (latent) variable, to gather all the unexplained variance. Here, Pz appears as a common source of causality for all voxels in the system, to account for the unexplained influence coming from F7, except for O1 and O2, which do not receive influenced from any other variable in the original system. This result is a beautiful example of the latent variable effect mentioned by Wong and Ozaki (2007). Note that Pz does not receive influence from any other variable.

**Figure 10:**
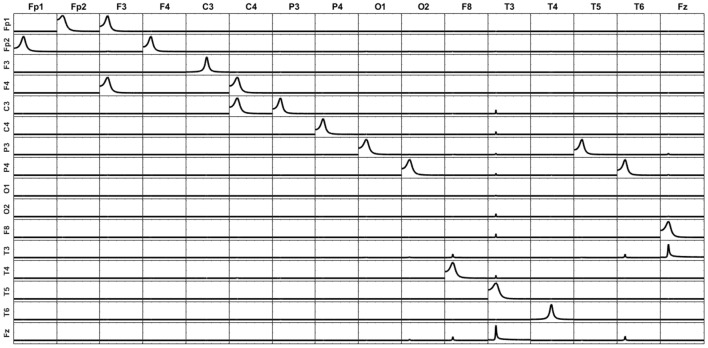
The unmixing procedure was carried out with the 17 point sources (including F7), but iCoh was computed based on the 16 sources resulting from removing F7. The variance due to F7 is then unknown for the iCoh measures. The connectivity patterns between all variables are preserved, except a bidirectional connection between Fz and T3. Note that this connection exists in the original system, mediated by F7, which has been removed. Small artifacts appear because the voxels without signal were set to random noise. If no noise is added to these voxels, the reconstruction does not show artifacts.

The third scenario for exploring the effect of incomplete information is keeping all the 17 variables of the system (including) for the unmixing procedure but calculating the iCoh between the 16 variables remaining after removing F7. Figure 10 shows the patterns of causality for this case. Compare this figure with Figure 9. In this case, almost no artifacts appear since F7 participated in the unmixing procedure. However, note that there is a bidirectional pattern of influence between Fz and T3. This is because in the original system, F7 sent information both to Fz and T3 (see Figure 6). When F7 is removed from the iCoh analysis, the *indirect* relationship that exists in the original system between Fz and T3, mediated by F7, appears now in iCoh measures as a **direct** relationship between them. This behavior is expected for measurements like iCoh and has nothing to do with the mixing effect.

The main conclusion from the results in Figures 8–10 is that, independently of the number of variables that will be included in the causality analysis, the unmixing procedure should be always carried out including the maximum admissible number of point sources in the model (containing, of course, those voxels to be subject to causal connectivity analysis). This number is always the number of electrodes minus one. This will guarantee the greater possible amount of unmixing of the signals to be submitted to the causal analysis.

### Surface ROI-based unmixing

**Figure 11:**
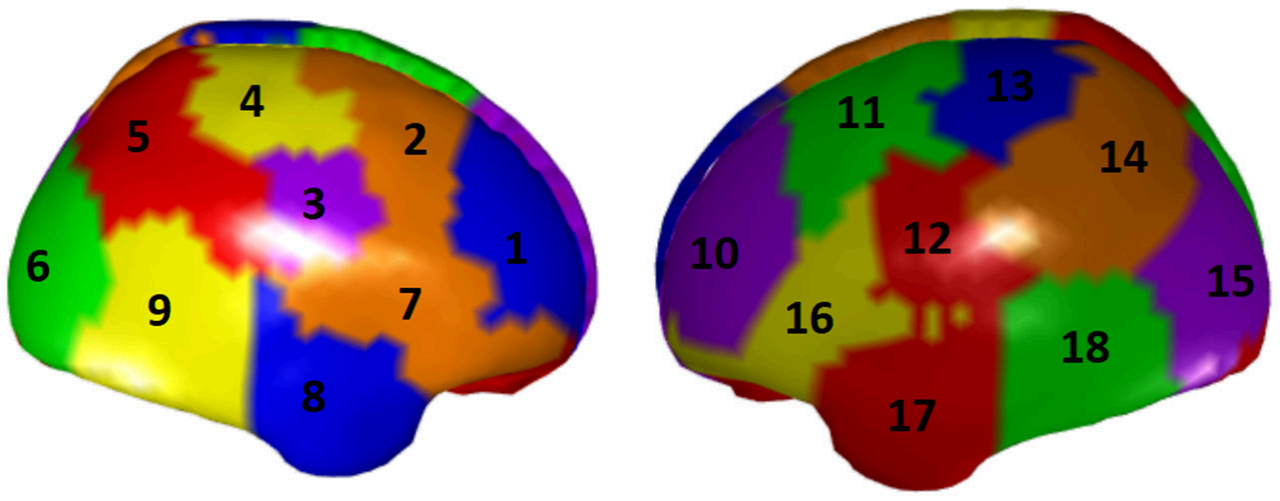
A parcellation of the cortex superficial grid in 18 regions. Each color represents a region, and the regions has been numbered consecutively.

To illustrate the ROI-based unmixing approach described in section Unmixing through regions on the cortex surface with known source directions with surface ROIs regions [i.e., the (**S2)** type of brain regions mentioned in section Unmixing through arbitrary regions with completely unknown source vectors], a new simulation is here added. We take a grid defined over the cortex surface of the MNI template and calculate a superficial lead field for it. Again, according to Equation (25) in section Unmixing through regions on the cortex surface with known source directions, to increase the number of independent local sources in the brain, the lead field is projected to the normal direction of each voxel. Then, we divide the external side of the surface into 18 disjoint regions, 9 regions in each hemisphere, as it is shown in Figure 11.

The simulation consisted in a source activity that is not constant over each region; specifically, for all voxels of Region 5 we created a Gaussian-shape source around a target value (value of the variable X1 in the system in Figure 1), and we added a random Gaussian noise, independent for each voxel and instant of time. The same procedure was used to assign the signal X2 of the system in Figure 1 to all voxels in Region 10, and the signal in X3 to all voxels in Region 17. The rest of the voxels of the grid were set to Gaussian white noise.

According to section Unmixing through arbitrary regions with completely unknown source vectors, to guarantee that the source estimates over the ROIs can be unmixed, i.e., the matrix **Q** in Equation (15) to be inversible, it is necessary to select the ROIs such that their centers are separated by a distance greater than the resolution of the inverse method. For this specific simulation, the condition number of the resulting matrix **Q** was 18.71, which indicates that the matrix is well conditioned.

**Figure 12:**
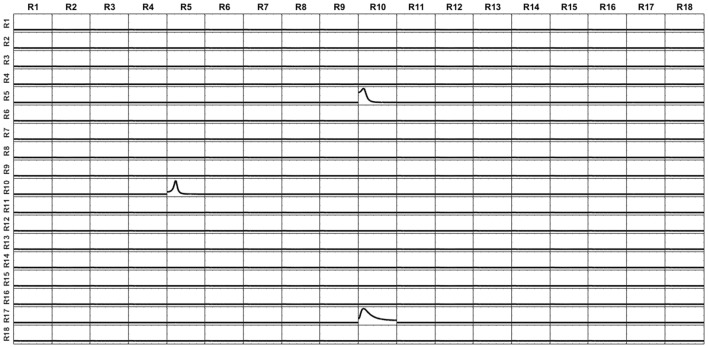
iCoh perfect reconstruction of the connectivity patterns simulated for the 18 surface regions, after application of the unmixing procedure for these surface regions, as described in section Unmixing through regions on the cortex surface with known source directions.

The voltage at the electrodes was obtained by solving the EEG forward problem from these currents, using the superficial, projected, lead field. Then, the current at the sources were reconstructed by solving sLoreta based on the generated voltage and the superficial lead field. The unmixing algorithm based on said 18 surface ROIs was applied, so obtaining the average unmixed signal for each ROI. For the 18 ROIs, the iCoh was calculated based on the unmixed reconstructed signals. Figure 12 shows the exact reproduction of the causality between the 18 ROIs for the reconstructed signals at the sources. There is a perfect reconstruction of the simulated patterns of information flow between the regions.

### An argument against the use of superficial lead fields for EEG connectivity analysis

In this section we analyze the negative effect in the connectivity analysis of using a superficial lead field (i.e., considering only voxels located in the surface of the cortex) when the sources that generate the voltage are not located only on the surface of the cortex.

To illustrate this, we used a volumetric lead field calculated over a grid that contained sources at the surface of the cortex, as well as sources deep in the gray matter.

We then selected the subset of voxels lying in the surface of the cortex and restricted the lead field to them. In this way, we have two lead fields: one for the volumetric grid and one for the voxels at the surface, where the superficial lead field contains a subset of the sources of the volumetric one. Both lead fields were projected to the normal direction to the cortex surface.

The next step was to create two scenarios, selecting two sets of voxels:

- In the first scenario, all the voxels belonging to the regions 5 and 10 of Figure 11 (which belong to the surface of the cortex, according to how the regions were created). Since the voxels are in the surface, they belong to both lead fields, the volumetric and the superficial ones.
- In the second scenario, for the regions 5 and 10 we found the centroids and select a small number of neighbors lying in the cortex **below the surface** (i.e., inner layer of the cortex). These voxels belong to the volumetric lead field but do not belong to the superficial one.

Voxels in Region 5 (or below, depending on the scenario) were connected to voxels in Region 10 (or below), in the two directions, i.e., from Region 5 to Region 10 and from Region 10 to Region 5.

Now, we used the volumetric lead field (which contains all the voxels, both at the surface and the volume) to generate the voltage at the electrodes, for solving the forward problem for the two scenarios. After this step, two sets of voltage signals are generated: one voltage for the signals located in the voxels of the surface and a second one for the signals located in the voxels of the inner layer.

Then, the next step was to use the two sets of generated voltages to estimate the currents back at the sources. For this purpose, the superficial lead field was used. Note that when the voltage is generated at the voxels in the inner layer, the superficial lead field does not contain information about these voxels; therefore, it will try to distribute this activity among the voxels in the surface. As inverse method, sLoreta was used. Finally, the estimated currents at the sources obtained by the two procedures were submitted to the unmixing method based on said 18 surface ROIs, as was described in section Unmixing through regions on the cortex surface with known source directions.

**Figure 13:**
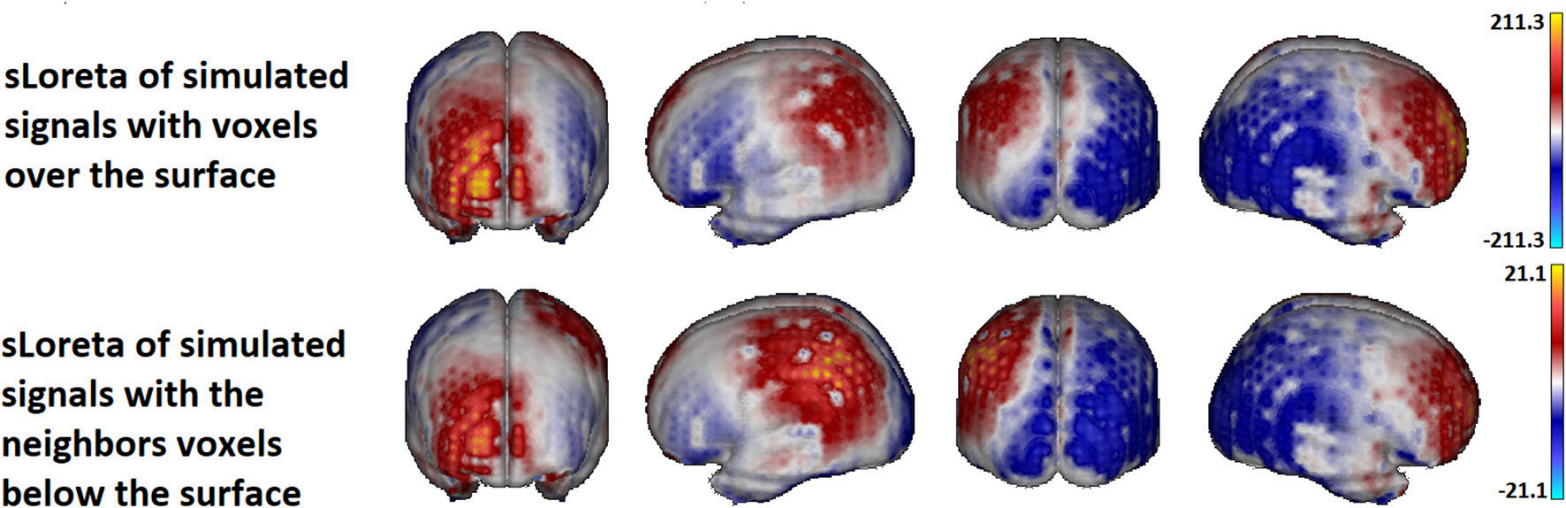
sLoreta solution using the superficial lead field. The **upper panel** shows the solution for the voltage generated with the set of voxels located in the surface. The **lower panel** shows the solution for the voxels lying in the volume, in the inner layer next to the voxels in the surface. The source localization is not affected by the position of the sources either at the surface or immediately below the surface voxels. Only the amplitude of the solution is smaller for the voxels in the inner layer.

Figure 13 shows the sLoreta solution for the two cases. Upper row contains the solution for the source voxels in the surface. Lower row contains the solution for the source voxels in the inner layer. The true solution is lying in the cortex below the regions 5 and 10 and is not shown but comparing with Figure 11 it is evident that the solutions coincide with the simulated sources. Although the amplitude of the solution for he voxels in the inner layer is 10 times smaller (a known effect of the Loreta family solutions for deep voxels), the topographic pattern of both solutions is the same. It means, the localization of the activity is not significantly affected, if the activity is not directly generated at the voxels of the surface but in the inner (deeper) neighbors, under the assumption that the currents on the cortex propagates to the surface in the perpendicular direction to the surface (i.e., in the normal direction).

**Figure 14:**
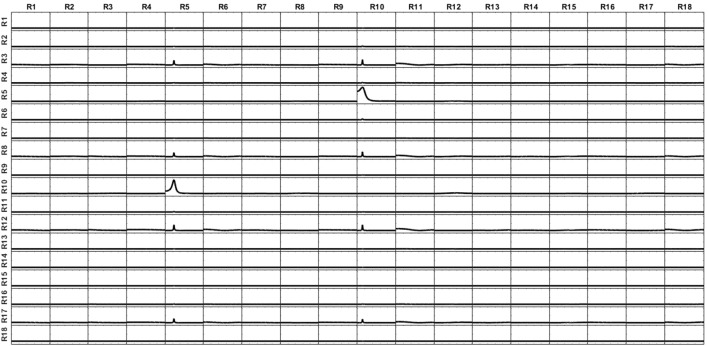
iCoh connectivity for the setting of current at the surface voxels reconstructed by the superficial lead field and sLoreta. The patterns are recovered with great accuracy; only some small artifacts randomly appear, due to the Gaussian white noise added to the simulated signals. Remember that in this simulation, the voxels at the surface are a subset of the voxels in the volume.

However, the connectivity patterns recovered by iCoh from the reconstructed signals at the sources are very different for the two scenarios. Figure 14 shows the patterns of connectivity for the currents at the sources estimated using the voxels in the surface (the first scenario a). Note that even when the signals have been reconstructed with the volumetric lead field, the iCoh patterns are completely preserved. A few number of small spurious artifacts appear between some isolated regions. These artifacts appear only when Gaussian white noise is added to the voxels in the volume. If no noise is added (a deterministic non-zero value is assigned to the voxels), the reconstruction is perfect.

**Figure 15:**
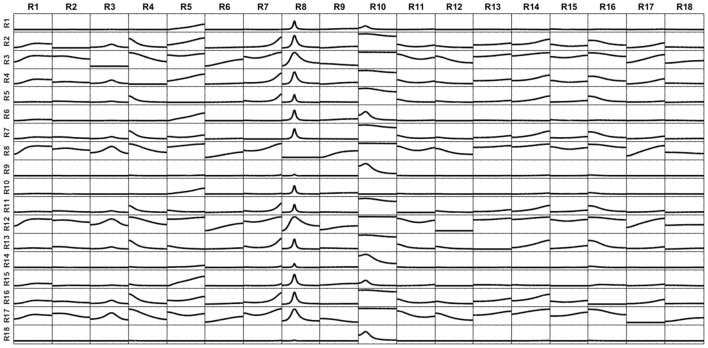
iCoh patterns of connectivity obtained from reconstructed currents at the cortex surface by using the superficial lead field when the voltage was generated by sources lying in the inner layer, bellow but close to the surface. The big effect of mixing in distorting the real connectivity pattern is because of the superficial lead field does not regard the voxels in the inner layer, hence surface-based unmixing lacks relevant information on inner voxels to successfully unmixed the superficial sLoreta source estimate.

On the contrary, the connectivity patterns for the signals reconstructed at the surface in the second scenario (the voltage was generated by voxels located in the inner layer) are totally distorted, even after applying the unmixing procedure. This is shown in Figure 15. This effect may be caused by the fact that the connectivity analysis is missing all the truly relevant variables in the system, namely the sources in the inner layer. This is an extreme example of Figure 10. In this case, the voxels in the surface are receiving influences from the voxels in the inner layer, which are unknown for the system analysis. Therefore, it is impossible successfully to apply an unmixing procedure based only on surface regions, since in this way the real variables are disregarded.

The conclusion of these simulations is that using superficial grids and lead fields may lead to wrong estimates of the brain connectivity at the sources if the real sources are not located at the surface. The problem is not sLoreta. It has been widely shown in this paper that after the unmixing procedure the connectivity at the sources is recovered in an almost perfect way. The problem is that solving the inverse problem at the surface ignores the real sources of the activity. The activities reconstructed for the sources located on the surface are already mixed due to the volume conduction problem; and, since only superficial sources are considered, the activity that arrives mixed at the surface, cannot be unmixed because of missing information present in the deep sources.

## Concluding remarks

The ROIs-based approach introduced in this paper for unmixing inverse solutions offers wide flexibility in specifying the family of ROIs that be adopted to suitably segment the brain in each particular experimental setting. These can be volumetric, surface or point regions of the brain, or even some combination of them.

The error when unmixing by this method, as is demonstrated by theoretical bounds and exemplified in simulations, depends on two main factors: (a) the exactness of the adopted segmentation for representing the true source field, and (b) the deviation of the resolution matrix from the identity. The first factor can be improved when the ROIs are selected taking into consideration relevant anatomical and functional knowledge about the underlying brain activity. It can be also enhanced by substantially increasing the number of regions in the brain segmentation model adopted, which in turn requires to increases the number of EEG recording electrodes.

The importance of this last issue to improve connectivity analysis is worth of be emphasized. The conclusion of section Point ROI-based unmixing with exact source models points out the importance of including the largest admissible number of ROIs in the unmixing procedure, even if not all of them will be later included in computing connectivity measures (such as iCoh). The largest admissible number of ROIs (variables) is always the number of electrodes minus one. This is a sound reason that enforces the need of increasing the number of electrodes.

The results shown in section Point ROI-based unmixing with exact source models of systems with more variables clearly advise against the use of superficial lead fields for brain connectivity analysis. The landscape of activation, i.e., the amplitude field of the source activity is not significantly affected by the use of superficial lead fields (at least when the activity comes from neighbor voxels located immediately under the cortex). However, the connectivity pattern associated with the flow of information arriving at the surface from inner voxels is completely misunderstood when only the voxels at the surface are regarded.

## Data availability

The simulations data and Matlab used in this work are freely available, under GNU license, at: doi: 10.6084/m9.figshare.6223778.

## Author contributions

RB elaborated the mathematical formulation, discussions of the methodology, validation of the simulations and substantial part of writing and editing of the manuscript; JB-B participated in the discussions of the methodology, wrote the programs, created the simulations and figures, and substantial part of writing and editing of the manuscript; RP-M participated in the mathematical formulation, discussions, validation of the simulations and revision of the manuscript.

### Conflict of interest statement

The authors declare that the research was conducted in the absence of any commercial or financial relationships that could be construed as a potential conflict of interest.

## Appendix

Let **j**(·)be any source field; **j**^0^(·), $\text{b}=\left({\text{b}}_{1}^{T},\ldots ,{\text{b}}_{L}^{T}\right)$ be defined by the Equations (11, 12); and $\hat{\text{b}}=\left({\hat{\text{b}}}_{1}^{T},\ldots ,{\hat{\text{b}}}_{L}^{T}\right)$ be the estimate of **b** obtained by solving the Equation (14).

Then, it holds the following bound for the size of the error $\text{b}-\hat{\text{b}}$:

(A1) $$\begin{array}{c} \|\text{b}-\hat{\text{b}}\|\leq \|{\text{Q}}^{-1}\|\|\delta \|, \end{array}$$

where ||·|| denotes the Euclidean norm of a vector or matrix, **Q** = (**Q**(*l,s*))_1 ≤ *l, s* ≤ *L*_, **Q**(*l,s*) is defined by , **Q**^−1^ is the inverse of the matrix **Q**, $\delta =\left(\delta_{1}^{T},\ldots ,\delta_{L}^{T}\right)$, and

(A2) $$\begin{array}{c} \delta_{l}=\underset{u\in B_{l}}{\sum }\underset{w\in B_{s}}{\sum }\text{R}\left(u,w\right)\left({\text{j}}^{0}\left(w\right)-\text{j}\left(w\right)\right). \end{array}$$

The inequality can be obtained as a straightforward consequence of the fact that

$$\begin{array}{l} \text{Q}\left(\text{b}-\hat{\text{b}}\right)=\delta . \end{array}$$

In turn, this last equation can be derived quite directly from the Eequations (14)–(15), (9), and (11–12).

Note that

(A3) $$\begin{array}{c} \|\delta \|\leq {\left\|\text{R}-diag\left(\text{R}\right)\right\|}_{\sup}\|{\text{j}}^{0}-\text{j}\|, \end{array}$$

where *diag*(**R**) denotes the diagonal of the matrix **R**, and

$$\begin{array}{l} {\left\|\text{C}\right\|}_{\sup}=\max\left\{\lfloor c_{ij}\rfloor :1\leq i,j\leq L\right\} \end{array}$$

for any matrix **C** = (c_ij_). Therefore, substituting (A3) into (A1), it is obtained

(A4) $$\begin{array}{c} \|\text{b}-\hat{\text{b}}\|\leq \|{\text{Q}}^{-1}\|\|{\text{j}}^{0}-\text{j}\|\underset{u\neq w}{\max}|\text{R}\left(u,w\right)|. \end{array}$$

This last equation shows that the error $\left\|\text{b}-\hat{\text{b}}\right\|$ of the estimate $\hat{\text{b}}$ is small if either ||**j**^0^−**j**|| or $\underset{u\neq w}{\max}|\text{R}\left(u,w\right)|$ are small, in the sense of the norms involved.

As said in section Unmixing through arbitrary regions with completely unknown source vectors, the condition for the inverse matrix **Q**^−1^to exist is that the minimum distance between all centers of the L ROIs is greater than the resolution of the inverse method used. In general, this results to be a well-conditioned matrix. For example, the condition number obtained for the case of simulation of 5 sources without measurement noise was 2.99, and for 5 sources with measurement noise it added was 47.94.

## References

[B1] Baccalá L. A. Sameshima K. (2001). Partial directed coherence : a new concept in neural structure determination. Biol. Cybernet. 84, 463–474. 10.1007/PL00007990 11417058

[B2] Baccala L. A. Sameshima K. Takahashia D. Y. (2007). Generalized partial directed coherence. Digit. Signal Process. 3, 163–166. 10.1109/ICDSP.2007.4288544

[B3] Bosch-Bayard J. Valdés-Sosa P. Virues-Alba T. Aubert-Vázquez E. John E. R. Harmony T.. (2001). 3D statistical parametric mapping of EEG source spectra by means of variable resolution electromagnetic tomography (VARETA). Clin. EEG 32, 47–61. 10.1177/155005940103200203 11360721

[B4] Chowdhury R. A. Lina J. M. Kobayashi E. Grova C. (2013). MEG source localization of spatially extended generators of epileptic activity: comparing entropic and hierarchical bayesian approaches. PLoS ONE 8:e55969. 10.1371/journal.pone.0055969 23418485 PMC3572141

[B5] Daunizeau J. Mattout J. Goulard B. Lina J.-M. Benali H. (2004). Data-driven cortex parcelling: a regularization tool for the EEG/MEG inverse problem, in 2nd IEEE International Symposium on Biomedical Imaging: Macro to Nano (IEEE Cat No. 04EX821) (Vol. 2) (Arlington, VA: IEEE), 1343–1346.

[B6] Evans C. D. Millst S. Brown E. Kelly R. Peters T. (1993). 3D statistical neuroanatomical models from 305 MRI volumes, in Proceedings of IEEE- Nuclear Science Symposium and Medical Imaging Conference, 1813–1817. Available online at http://www.citeulike.org/user/nguizard/article/7253872

[B7] Farahibozorg S.-R. Henson R. N. Hauk O. (2018). Adaptive cortical parcellations for source reconstructed EEG/MEG connectomes. NeuroImage 169, 23–45. 10.1016/j.neuroimage.2017.09.009 28893608 PMC5864515

[B8] Giacometti P. Perdue K. L. Diamond S. G. (2014). Algorithm to find high density EEG scalp coordinates and analysis of their correspondence to structural and functional regions of the brain. J. Neurosci. Methods 229, 84–96. 10.1016/j.jneumeth.2014.04.020 24769168 PMC4071772

[B9] Grech R. Cassar T. Muscat J. Camilleri K. P. Fabri S. G. Zervakis M.. (2008). Review on solving the inverse problem in EEG source analysis. J. NeuroEng. Rehabilitat. 5:25. 10.1186/1743-0003-5-25 18990257 PMC2605581

[B10] Hämäläinen M. S. Ilmoniemi R. J. (1994). Interpreting magnetic fields of the brain: minimum norm estimates. Med. Biol. Eng. Comput. 32, 35–42. 10.1007/BF02512476 8182960

[B11] Hedrich T. Pellegrino G. Kobayashi E. Lina J. M. Grova C. (2017). Comparison of the spatial resolution of source imaging techniques in high-density EEG and MEG. NeuroImage 157, 531–544. 10.1016/j.neuroimage.2017.06.022 28619655

[B12] Hoke M. Lutkenhoner B. Pantev C. (1990). Comparison between different methods to approximate an area of the human head by a sphere, in Advances in Audiology, Auditory-Evoked Magnetic Fields and Electric Potentials, eds Hoke M. Grandori F. Romani G. L. (Basel: Karger), 165–193.

[B13] Hutchison J. L. Hubbard N. A. Brigante R. M. Turner M. Sandoval T. I. Hillis G. A. J.. (2014). The efficiency of fMRI region of interest analysis methods for detecting group differences. J. Neurosci. Methods 226, 57–65. 10.1016/j.jneumeth.2014.01.012 24487017 PMC4000065

[B14] Jatoi M. Begum T. Shahid A. (2014). Brain source localization using EEG signals, in EEG/ERP Analysis: Methods and Applications, 1st Edn, eds Kamel N. Malik A. S. (Boca Raton, FL: CRC Press), 91–122.

[B15] Lapalme E. Lina J.-M. Mattout J. (2006). Data-driven parceling and entropic inference in MEG. NeuroImage 30, 160–171. 10.1016/j.neuroimage.2005.08.067 16426867

[B16] Pascual-Marqui R. Biscay R. J. Bosch-Bayard J. Lehmann D. Kochi K. Kinoshita T.. (2014). Assessing direct paths of intracortical causal information flow of oscillatory activity with the isolated effective coherence (iCoh). Front. Hum. Neurosci. 8:448. 10.3389/fnhum.2014.00448 24999323 PMC4064566

[B17] Pascual Marqui R. D. (1999). Review of methods for solving the EEG inverse problem. Int. J. Biomagn. 1, 75–86.

[B18] Pascual-Marqui R. D. (2002). Standardized low resolution brain electromagnetic tomography (sLORETA): technical details. Methods Find. Exp. Clin. Pharmacol. 24 (Suppl. D), 5–12. 12575463

[B19] Pascual-Marqui R. D. Lehmann D. Koukkou M. Kochi K. Anderer P. Saletu B.. (2011). Assessing interactions in the brain with exact low-resolution electromagnetic tomography. Philos. Trans. R. Soc. 369, 3768–3784. 10.1098/rsta.2011.0081 21893527

[B20] Pascual-Marqui R. D. Michel C. M. Lehmann D. (1994). Low resolution electromagnetic tomography: a new method for localizing electrical activity in the brain. Int. J. Psychophysiol. 18, 49–65. 10.1016/0167-8760(84)90014-X 7876038

[B21] Poldrack R. A. (2007). Region of interest analysis for fMRI. Soc. Cogn. Affect. Neurosci. 2, 67–70. 10.1093/scan/nsm006 18985121 PMC2555436

[B22] Riera J. J. Fuentes M. E. (1998). Electric lead field for a piecewise homogeneous volume conductor model of the head. IEEE Trans. Biomed. Eng. 45, 746–753. 10.1109/10.678609 9609939

[B23] Wong K. F. K. Ozaki T. (2007). Akaike causality in state space. Instantaneous causality between visual cortex in fMRI time series. Biol. Cybern. 97, 151–157. 10.1007/s00422-007-0165-1 17579884

